# Protein changes in the retina following experimental retinal detachment in rabbits

**Published:** 2011-10-08

**Authors:** Nakul Mandal, Geoffrey P. Lewis, Steven K. Fisher, Steffen Heegaard, Jan U. Prause, Morten la Cour, Henrik Vorum, Bent Honoré

**Affiliations:** 1 Eye Pathology Section, Department of Neuroscience and Pharmacology, University of Copenhagen, Denmark; 2 Department of Biomedicine, Aarhus University, Denmark; 3 Department of Ophthalmology, Glostrup Hospital, University of Copenhagen, Denmark; 4 Department of Ophthalmology, Aalborg Hospital, Aarhus University Hospital, Denmark; 5 Neuroscience Research Institute, University of California, Santa Barbara, CA; 6 Department of Molecular, Cellular and Developmental Biology, University of California, Santa Barbara, CA

## Abstract

**Purpose:**

Retinal detachment leads to the widespread cellular remodeling of the retina. The purpose of this study was to identify protein changes that accompany these cellular alterations by comparing the proteomic profiles of sham and experimentally detached rabbit retina. Elucidation of the proteins most dramatically affected by retinal detachment would add further understanding to the pathophysiology of this condition, and potentially identify therapeutic targets useful in preventing the deleterious effects of detachment, including photoreceptor cell death and the activation of non-neuronal microglial and Müller cells.

**Methods:**

Retinal detachments were induced in the right eyes of six New Zealand Red pigmented rabbits. Sham surgery was performed in the right eyes of six other rabbits that were used as controls. At seven days, the eyes were enucleated and the retinal tissue was harvested. The individual retinal samples were subjected to high resolution two-dimensional polyacrylamide gel electrophoresis. Differentially expressed protein spots were processed for identification by liquid chromatography-tandem mass spectrometry. Further investigation was undertaken with western blotting, and immunocytochemical studies on a further set of four sham and four detached retinas.

**Results:**

Eighteen protein spots were found to be at least twofold differentially expressed between the sham and detached retinas. These protein spots were identified as: vimentin; tubulin β-2C; fragments of α-enolase; fructose-bisphosphate aldolase A; ATP synthase subunit β; mitochondrial creatine kinase; N-terminal fragments of albumin; prohibitin; and transducin-β_1_.

**Conclusions:**

The differentially expressed proteins determined in this study may play an important role in the cellular responses of the retina after its detachment, subsequent ability to recover following surgical reattachment, as well as in serious complications such as subretinal fibrosis and proliferative vitreoretinopathy.

## Introduction

There has been great advancement in the practice of retinal surgery since Jules Gonin’s pioneering work on retinal detachment repair from the early twentieth century [1]. Today, anatomical reattachment of the neurosensory retina following rhegmatogenous retinal detachment is successfully achieved in approximately 90% of cases following primary surgery [2]. However, proliferative vitreoretinopathy (PVR), which is estimated to occur in 5–10% of cases of rhegmatogenous retinal detachment, remains the main cause of failed reattachment surgery [3-6]. PVR is an unwelcome wound healing process of the retina, which is characterized by the proliferation of numerous cell types, including retinal pigment epithelial (RPE) cells, Müller cells, astrocytes, immune cells, and hyalocytes that result in the formation of retinal and vitreal cicatricial membranes. Rhegmatogenous retinal detachment results in the loss of the close intercellular relationship between the photoreceptors and RPE cells, and their consequent exposure to the vitreous. RPE cells are thus induced to proliferate and migrate into the subretinal space and vitreous cavity where they are postulated to undergo epithelial-to-mesenchymal transition with an ability for extracellular matrix (ECM) production and contractility [5,7-11]. The membranes formed from the proliferation and growth of hypertrophied Müller cells into the subretinal space and vitreous act as a scaffold on which other cells can migrate, proliferate, and synthesize ECM constituents, and also offer support for the growth of neurites originating from horizontal and ganglion cells [5,12-17]. The presence of subretinal scarring can hinder the reestablishment of the interface between the photoreceptors and RPE, preventing the recovery of vision after surgical reattachment [18], while contraction of periretinal membranes can apply deleterious tension on the retina, causing retinal folding, the opening of old retinal breaks, and the formation of new ones, which may result in tractional retinal detachment [3,19,20].

Despite the elucidation of the role of numerous cells and growth factors involved in the pathogenesis of PVR, there is presently no effective pharmacological agent for the treatment of this condition in patients [3,21-25].

In an effort to further understand the biochemical and cellular remodelling processes occurring in retinal detachment, subretinal fibrosis and PVR, with the ultimate goal of finding novel biomarkers and therapeutic targets we performed the first proteomic analysis of the retina in an animal model of this condition, whose well characterized retinal changes [26,27] have been shown to share many features with the human form of the disease [28-31]. Indeed, it is the proteins as the effectors of gene expression that will ultimately determine the pathophysiological changes in the retina following detachment and its ability to functionally recover following surgical reattachment [32].

## Methods

### Retinal detachment surgery

Six New Zealand Red pigmented rabbits were anesthetized using an intramuscular injection of xylazine and ketamine (6.7 and 33.3 mg/kg, respectively). The pupils were dilated with topical drops of atropine and tropicamide (1% solutions). A fine custom-pulled glass micropipette with a tip diameter of approximately 100 µm was connected via tubing to a 10 ml syringe attached to an infusion pump. The syringe contained a solution of sodium hyaluronate (Healon, 0.25% in a balanced salt solution; Pharmacia, NJ). Using an ophthalmic operating microscope, the pipette, which was attached to a micromanipulator, was carefully advanced through an incision in the sclera and pars plana of the right eye, avoiding the lens. A corneal contact lens was used to better visualize the interior of the eye. As soon as the pipette tip was observed touching the retina, the infusion pump was activated under the control of a foot pedal, which started an infusion of Healon solution into the subretinal space. Healon was necessary to prevent spontaneous retinal reattachment. After approximately 50% of the inferior neural retina was detached, the pipette was withdrawn and the scleral incision was closed with an 8–0 nylon suture. Sham surgery, which involved making the scleral incision and inserting the pipette into the vitreous cavity without disruption of the retina, was performed in the right eyes of six other rabbits that were used as controls. No signs of raised intraocular pressure (e.g., hardness of the eye, inflammation) were noted during or after the surgical procedures. Seven days after the surgery, the twelve rabbits were euthanized with Euthasol (120 mg/kg; Butler Schein, Dublin, OH), the eyes were enucleated, and the corneas and lenses were removed. Thereafter, the inferior detached and sham retinas were peeled from the underlying RPE in the respective eyes and were immediately snap frozen in liquid nitrogen within separate vials. Particular care was taken to avoid contamination of the neurosensory retina samples with RPE cells, choroid or vitreous. The samples were stored at −80 °C until further use.

The animal experiments undertaken in this study conformed to the National Institutes of Health Animal Care and Use Committee protocols, the ARVO statement for the Use of Animals in Ophthalmic and Vision Research, and the guidelines of the Animal Resource Centre, University of California, Santa Barbara.

### Protein extraction

Retinal tissue was homogenized and dissolved in a lysis buffer containing 9 M urea, 2% (v/v) Triton X-100, 2% (v/v) immobilized pH gradient (IPG) buffer (pH 3–10 nonlinear), and 2% (w/v) dithiothreitol (DTT). The total protein content in each retinal sample was determined with the Non-Interfering Protein Assay (Calbiochem, San Diego, CA). The extracted protein samples were stored at −80 °C.

### Two-dimensional gel electrophoresis

The first dimension isoelectric focusing (IEF) was performed using pH 3–10 nonlinear 18 cm IPG strips (GE Healthcare, Chalfont St. Giles, Buckinghamshire, UK). Each IPG strip was rehydrated for 20 h at room temperature in 200 µl lysis buffer each containing 40 µg protein from individual retinal samples, and 150 µl rehydration buffer (8 M urea, 2% (w/v) 3-[(3-cholamidopropyl)dimethylammonio]-1-propanesulphonate (CHAPS), 0.3% (w/v) DTT and 2% (v/v) IPG buffer), using the Immobiline DryStrip Reswelling Tray (GE Healthcare). IEF was performed on a Multiphor II Electrophoresis System (GE Healthcare) at 500 V for 5 h, 3500 V for 5 h, and 3500 V for 9.5 h in a gradient mode at 17 °C using a MultiTemp III Thermostatic Circulator (GE Healthcare). Prior to the second dimension sodium dodecyl sulfate (SDS) Polyacrylamide gel electrophoresis (PAGE), the IPG strips were equilibrated twice; first for 10 min under gentle agitation in 20 ml of equilibration buffer (0.6% (w/v) Tris-HCl, pH 6.8, 6 M urea, 30% (v/v) glycerol, 1% (w/v) SDS, and 0.05% (w/v) DTT); second using, 4.5% (w/v) iodoacetamide and bromophenol blue. For the second dimension, the equilibrated IPG strips were transferred to 12% polyacrylamide gels. Electrophoresis was then performed vertically at a maximum voltage of 50 V for approximately 20 h.

### Protein staining

The proteins in the gels were visualized by silver staining optimized for high sensitivity protein identification by mass spectrometry [33]. Briefly, individual gels were fixed overnight in 50% (v/v) ethanol, 12% (v/v) acetic acid, and 0.0185% (v/v) formaldehyde. Following washing 3 times for 20 min in 35% (v/v) ethanol and pretreatment for 1 min in 0.02% (w/v) Na_2_S_2_O_3_.5H_2_O, the gels were rinsed in water and stained in 0.2% (w/v) AgNO_3_, and 0.028% (v/v) formaldehyde for 20 min. Further rinsing with water was performed before development in 6% (w/v) Na_2_CO_3_, 0.0185% (v/v) formaldehyde, and 0.0004% (w/v) Na_2_S_2_O_3_.5H_2_O, for approximately 3 min. Finally, development was arrested in a fixative solution of 40% (v/v) ethanol and 12% (v/v) acetic acid.

### Image analysis

Dried silver-stained gels were scanned in a transmission mode using a GS-710 Calibrated Imaging Densitometer (Bio-Rad, Hercules, CA). The protein spots were analyzed using PDQuest (Bio-Rad) software, which designated a volume to each spot proportional to the amount of protein. All well separated and clearly focused spots that were differentially expressed at least 2.0-fold (Mann–Whitney *U* test, p<0.05), between the sham and detached retina groups were selected for identification by liquid chromatography-tandem mass spectrometry (LC-MS/MS).

### Protein identification

The proteins were excised from the gels, subjected to in-gel tryptic digestion and the peptide samples were analyzed by LC-MS/MS as previously described [34]. In short, digested peptides were separated on an inert nano LC system (LC Packings, San Francisco, CA) connected to a Q-Tof Premier mass spectrometer (Waters, Milford, MA). Spectra were obtained using MassLynx 4 SP4 (Waters). Raw data were processed using ProteinLynx GlobalServer 2.1 (Waters). The processed data were used to search the total part of the Swiss-Prot database using the online version of the Mascot MS/MS Ions Search facility (Matrix Science, Ltd.) [35]. Searching was performed with doubly and triply charged ions with up to two missed cleavages, a peptide tolerance of 50 ppm, one variable modification, Carbamidomethyl-C, and a MS/MS tolerance of 0.05 Da. Contaminating peptides such as keratin, trypsin, bovine serum albumin, and all peptides from previous samples were disregarded. At least one ‘bold red’ peptide was required in the search. Individual peptide ions scores above approximately 36 indicated identity or extensive homology, giving a less than 5% probability that the observed match was a random event. All mass spectra were manually verified.

### Western blotting

Twenty micrograms of protein from each retinal sample was loaded and separated upon Novex 10%–20% gradient Tris-Glycine polyacrylamide gels (Invitrogen Corporation, Carlsbad, CA). After transfer by electro-elution to nitrocellulose Hybond-C Extra membranes, blots were blocked overnight with 5% skimmed milk in 80 mM Na_2_HPO_4_, 20 mM NaH_2_PO_4_, 100 mM NaCl, and 0.05% Tween-20 (PBST), pH 7.5, and this was followed by incubation with the appropriate primary antibodies (Table 1). After washing, the membranes were incubated with the appropriate horseradish peroxidase (HRP)-conjugated secondary antibodies: P0260 mouse (1:1000), P0449 goat (1:2000), and P0163 sheep (1:1000; DAKO, Glostrup, Denmark). Proteins were visualized using the enhanced chemiluminescence system (GE Healthcare), with an imaging system (Fujifilm LAS-3000; Tokyo, Japan), and were quantified with the integrated Multi Gauge software (Fujifilm).

**Table 1:** Primary antibodies used in this study along with their source and dilutions.

**Antibody**	**Clonality/Immunogen**	**Species and antibody class**	**Manufacturer**	**WB antibody dilution**	**ICC antibody dilution**
Tubulin β2	Monoclonal/human C-terminus peptide	Mouse IgG	Abcam, UK	ND	1:100
α-enolase	Monoclonal/recombinant full length human protein	Mouse IgG	Abcam, UK	1:2000	1:100
ATP synthase subunit β	Monoclonal/whole human heart mitochondria	Mouse IgG	Abcam, UK	1:2000	1:100
Albumin	Polyclonal/rabbit serum albumin	Sheep IgG	Genway Biotech, Inc., CA	1:5000	1:100
Vimentin	Polyclonal/recombinant full length hamster protein	Chicken IgY	Millipore, MA	ND	1:2000
Prohibitin	Monoclonal/recombinant full length human protein	Mouse IgG	Abcam, UK	NAR	ND
Creatine kinase, mitochondrial	Polyclonal/human C-terminus peptide	Goat IgG	Santa Cruz Biotech, Inc., CA	NAR	ND
Aldolase A	Polyclonal/rabbit muscle aldolase	Goat IgG	Abcam, UK	NAR	ND
G-protein β1	Polyclonal/human internal region peptide	Goat IgG	Santa Cruz Biotech, Inc., CA	NAR	ND
Isotype control	Polyclonal	Mouse IgG	Abcam, UK	ND	1:100
Isotype control	Polyclonal	Sheep IgG	Abcam, UK	ND	1:100
Isotype control	Polyclonal	Chicken IgY	Abcam, UK	ND	1:100
Isotype control	Polyclonal	Goat IgG	Abcam, UK	ND	1:100

Abbreviations: WB represents western blotting; ICC represents immunocytochemistry; NAR represents no adequate/specific reaction; ND represents not determined.

### Immunocytochemistry

Four additional New Zealand Red pigmented rabbits underwent retinal detachment induction while four others underwent sham surgery as described above. The eyes were fixed in 4% paraformaldehyde for a minimum of 24 h. Four detached and four sham inferior retinal pieces of approximately 4 mm^2^ were embedded in 5% low-melt agarose, sectioned at 100 µm using a vibratome, and were placed in plastic micro-beakers for the antibody processing. They were first incubated for 24 h in normal donkey serum (1:20) in PBS, 0.5% BSA, 0.1% Triton X-100, and 0.1% azide (PBTA) after which they were incubated (for 24 h) in the primary antibodies that were verified at the correct molecular mass on western blotting (Table 1). Following rinsing in PBTA (3×5 min, 1×1 h rinsing steps), appropriate secondary antibodies for mouse, goat, chicken, and sheep (1:200 in PBTA; Jackson ImmunoResearch, West Grove, PA) were applied for 24 h. Control experiments were performed using isotype antibodies in place of the primary antibodies, followed by the appropriate secondary antibodies (Table 1). Controls were also undertaken by omitting the primary antibodies and using the appropriate secondary antibodies alone. Sections were further rinsed, mounted on glass slides, and viewed using a laser scanning confocal microscope (Olympus FluoView 1000; Center Valley, PA). The resulting images represented a projection of five z-planes, which were collected at 0.5 µm intervals. Experimental detachment and sham retinas were viewed during the same imaging session with constant intensity and black level settings on the microscope.

## Results

### Differential protein expression profiles of sham and detached neural retina

**Figure 1:**
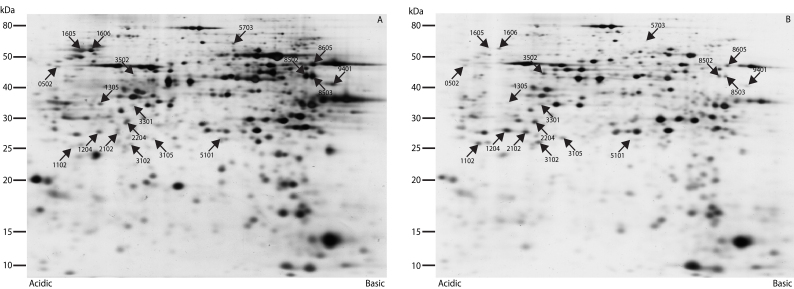
2D-PAGE analysis of the rabbit retina. Eighteen protein spots were found to be significantly and at least twofold differentially expressed between the sham (**A**) and detached retina (**B**). The gels are representative of the two groups.

Up to approximately 800 protein spots were clearly resolved by two-dimensional PAGE (2D-PAGE; Figure 1). The overall protein expression profiles of the twelve retinas were quite similar. Eighteen protein spots were found to be at least twofold differentially expressed between the sham and detached retinas. Nine protein spots were upregulated and nine were downregulated in the detached retina group. Fifteen of the eighteen protein spots were successfully identified using tandem mass spectrometry, as given in Table 2 and Appendix 1. Three spots eluded identification despite repeat processing. The nine upregulated spots were identified as: vimentin (spot 0502); fragments of α-enolase (spots 1102, 1204, 2102, and 2204); N-terminal fragments of albumin (spots 3102, 3105, and 3502); and prohibitin (spot 3301). Of the nine downregulated spots, we identified 1305 as transducin-β_1_; 1605 as tubulin β-2C; 1606 as ATP synthase subunit β; 8502 and 8503 as creatine kinase; and 9401 as fructose-bisphosphate aldolase A. Spots 5101, 5703, and 8605 could not be identified (Table 2).

**Table 2:** Mass spectrometric identification of the 2D-PAGE protein spots differentially expressed in the rabbit retina.

**Spot number**	**Protein name/Swiss-Prot entry** **(Full chain length)**	**Theoretical pI;*M*_r_ kDa**	**Fold change RD: Sham**	**Biological processes**
1204	**α-enolase** (434) ENOA_BOVIN, fragment	6.99;47.0	2.24	Glycolysis; Plasminogen activation; Transcription; Transcription regulation
2204/2102/1102	ENOA_MOUSE, fragment	6.99;47.0	2.28/2.41/2.42	
0502	**Vimentin** (466) VIME_HUMAN	5.06;53.5	2.51	Cell motion
3105/3102/3502	**Albumin** (608) ALBU_RABIT, N-terminal fragment	5.67;66.5	2.66/2.95/3.19	Transport; Cellular response to starvation; Maintenance of mitochondrial location; Negative regulation of apoptosis; Hemolysis of symbiont of host erythrocytes
3301	**Prohibitin** (272) PHB_HUMAN	5.57;29.8	2.72	Negative regulation of cell proliferation; Negative regulation of transcription; Regulation of apoptosis; Signal transduction; Histone deacetylation
9401	**Fructose-bisphosphate aldolase A** (364) ALDOA_RABIT	8.39;39.3	0.49	ATP biosynthetic process; Fructose 1,6-bisphosphate metabolic process; Regulation of cell shape; Actin filament organization; Striated muscle contraction; Muscle maintenance
1305	**Transducin-β1** (340) GBB1_HUMAN	5.60;37.2	0.48	Hormone-mediated signaling; Muscarinic acetylcholine receptor signaling; Signal transduction; Ras protein signal transduction
1606	**ATP synthase subunit β,** **mitochondrial** (528) ATPB_BOVIN	5.00;51.8	0.41	Protein transport; ATP synthesis coupled protein transport; Regulation of intracellular pH; Angiogenesis
8502/8503	**Creatine kinase, ubiquitous** **mitochondrial** (416) KCRU_BOVIN	7.31;43.1	0.38/0.35	Creatine metabolic process
1605	**Tubulin β-2C chain** (447) TBB2C_HUMAN	4.79;49.8	0.18	Cell motion; Microtubule-based movement; Natural killer cell mediated cytotoxicity; Protein polymerisation

Protein spots 5101, 5703, and 8605 with fold changes of 0.48, 0.43, and 0.38 respectively could not be identified. Biological processes were taken from the Gene Ontology Consortium. Abbreviation: RD represents retinal detachment.

### Immunological verification of the differential protein expression

The upregulation of vimentin and overall downregulation of tubulin β-2C (though increased expression within the remaining inner retina) following retinal detachment is in accordance with previous observations obtained by western blotting and immunocytochemistry [12,36], thereby confirming the validity of the current methodological approaches.

**Figure 2:**
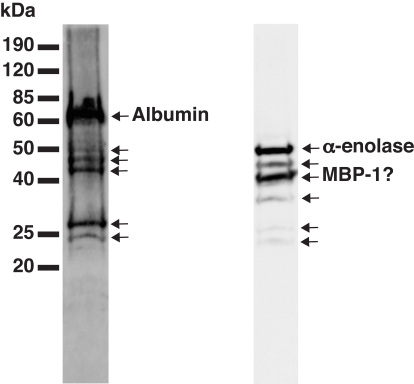
Western blot analysis of sham rabbit retina developed with anti-albumin and anti-α-enolase. The labeled arrows relate to the respective full length proteins. Unlabeled arrows may indicate the specific cleavage fragments, and judging by the approximate molecular mass it appears that some of these weaker bands may correspond with the differentially expressed 2D-PAGE protein spots. Abbreviation: MBP-1 represents Myc-binding protein-1.

Among the upregulated proteins, the molecular mass of vimentin (spot 0502) and prohibitin (spot 3301) as observed from the 2D-PAGE analysis are in reasonable agreement with the theoretical mass, suggesting that they are not fragments (Table 2). Three spots (3102, 3105, and 3502) were found to be N-terminal fragments of albumin. The approximate molecular mass of these fragments were between 25 kDa and 45 kDa. The presence of several fragments of albumin was verified by western blotting using a polyclonal antibody (Figure 2). The anti-albumin antibody recognizes a strong uncleaved albumin band together with several fragments of lower intensity and lower molecular mass. Judging by the approximate molecular mass, some of the weaker bands on the western blot correspond to the 2D-PAGE identified spots. An analogous pattern was found with α-enolase, which was detected as fragments in four spots (1102, 1204, 2102, and 2204) with molecular mass between 25 and 30 kDa on the 2D-PAGE analysis. This pattern is in accordance with a western blot analysis, revealing relatively strong bands at approximately 40 kDa and 50 kDa, together with several weaker bands between 25 kDa and 50 kDa (Figure 2). As with albumin, some of the weaker bands on the α-enolase western blot may correspond with the spots identified by 2D-PAGE.

**Figure 3:**
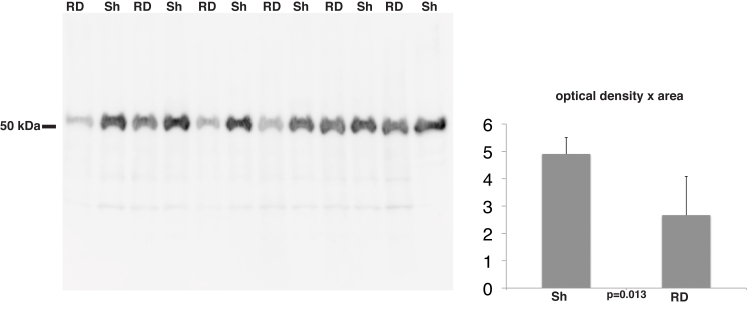
Western blot analysis of rabbit retinas developed with anti-ATP synthase subunit β. The histogram shows the mean densitometry of the antibody-antigen reaction. Mann–Whitney *U* test showed a significant decrease in the levels of ATP synthase subunit β with RD. Abbreviations: RD represents retinal detachment, Sh represents sham.

Among the downregulated spots we identified five proteins with observed molecular mass that are in reasonable agreement with their theoretical molecular mass (Table 2). Thus, these proteins do not seem to be fragments. We used a specific antibody against ATP synthase subunit β, which resulted in a western blot with reasonably strong distinct bands that were quantifiable (Figure 3). Quantification of the bands verified that ATP synthase subunit β is significantly downregulated in detached retina (Figure 3).

**Figure 4:**
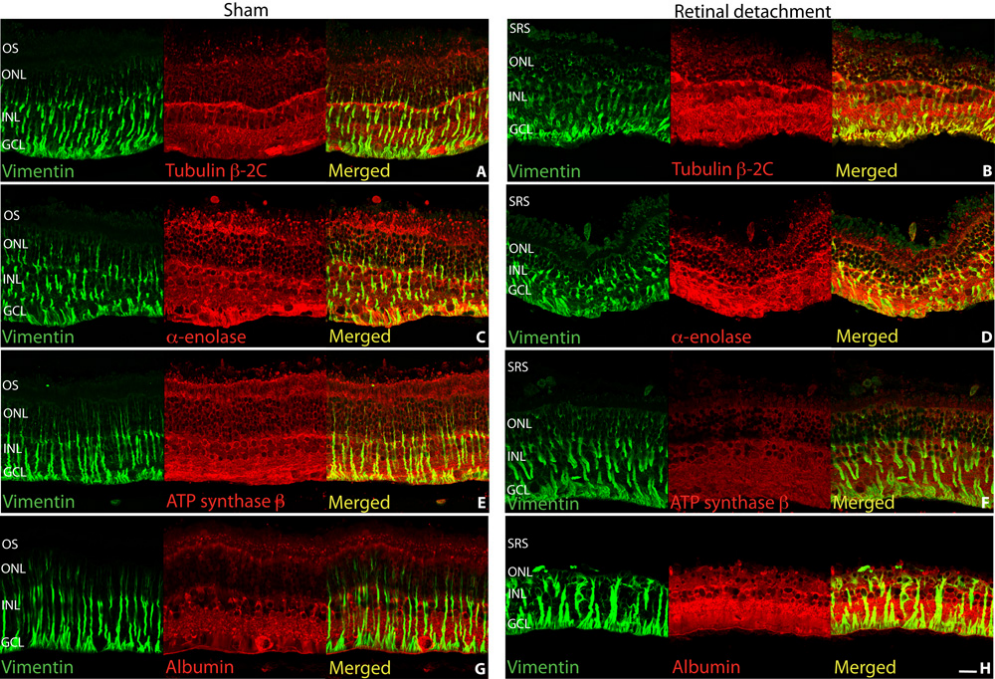
Laser scanning confocal images of sham (**A**, **C**, **E**, **G**) and detached (**B**, **D**, **F**, **H**) rabbit retina labeled with antibodies for vimentin (**A**-**H**, green), tubulin β-2C (**A**, **B**), α-enolase (**C**, **D**), ATP synthase (**E**, **F**), and albumin (**G**, **H**) all in red. Colocalization of the respective proteins in the Müller cells is suggested by greenish yellow to yellow color. Abbreviations: OS represents outer segments; ONL represents outer nuclear layer; INL represents inner nuclear layer; GCL represents ganglion cell layer; SRS represents subretinal space. Scale bar 20 µm.

**Figure 5:**
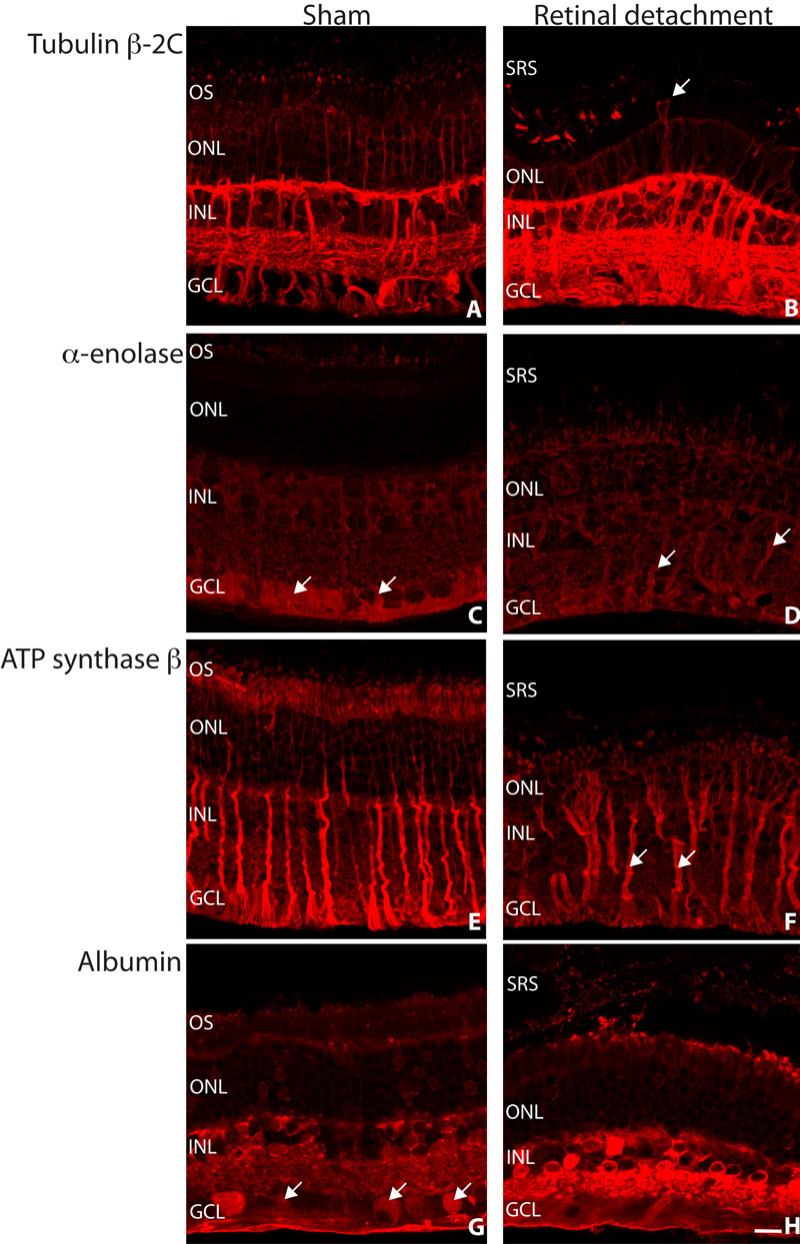
Laser scanning confocal images of sham (**A**, **C**, **E**, **G**) and detached (**B**, **D**, **F**, **H**) rabbit retina labeled with antibodies for tubulin β-2C (**A**, **B**), α-enolase (**C**, **D**), ATP synthase β (**E**, **F**), and albumin (**G**, **H**) all in red. Müller cells are often observed extending into the subretinal space following retinal detachment (**B**, arrow). Labeling for α-enolase was concentrated in the Müller cell endfeet region in sham retina, which is possibly redistributed within this cell type following retinal detachment (**C**, **D**, arrows). The Müller cells remained relatively brightly labeled for ATP synthase subunit β following retinal detachment (**F**, arrows). Variable albumin labeling of the ganglion cells was observed in sham retina (**G**, arrows). The albumin labeling that occurred intracellularly was shown to increase in intensity following retinal detachment (**G**, **H**). Abbreviations: OS represents outer segments; ONL represents outer nuclear layer; INL represents inner nuclear layer; GCL represents ganglion cell layer; SRS represents subretinal space. Scale bar 20 µm.

We further analyzed the expression pattern of selected proteins in the retina by immunocytochemistry as shown in Figure 4 and Figure 5. Vimentin was labeled in the Müller cells extending from the inner limiting membrane to the outer limiting membrane of the sham retina (Figure 4A,C,E,G). In detached retina, the Müller cell hypertrophy is accompanied by an increased expression of intracellular vimentin and more labeling into the outer retina (Figure 4B,D,F,H).

Labeling of sham retina reflected cytoskeletal tubulin β-2C. We found strong labeling of ganglion, Müller and horizontal cells, and of ganglion cell dendritic processes in the inner plexiform layer (IPL). Weaker labeling was found in the inner nuclear layer (INL) and outer nuclear layer (ONL; Figure 4A, Figure 5A). In the detached retina, we observed a dramatic increase in tubulin β-2C staining within the inner retina and Müller cells (Figure 4B, Figure 5B). Loss of the photoreceptor cell layer after detachment is likely to account for the overall decrease in tubulin β-2C found with the 2D-PAGE analysis [36] (Table 2). Müller cells labeled for tubulin β-2C can often be seen extending beyond the outer limiting membrane and into the subretinal space following retinal detachment (Figure 5B arrow).

Labeling for α-enolase in the sham retina showed the ganglion cell layer (GCL) and Müller cell endfeet region most strongly labeled (Figure 4C, Figure 5C arrows) with weaker intensity elsewhere in the retina. In the detached retina, we found overall increased labeling with the Müller cells becoming the most noticeably labeled (Figure 4D).

Antibodies directed against ATP synthase subunit β predominantly labeled Müller cells, the inner segments of photoreceptors with labeling throughout in the sham retina (Figure 4E, Figure 5E). In the detached retina we found decreased overall ATP synthase subunit β labeling (Figure 4F, Figure 5F) with the exception of the Müller cells, which remained strongly labeled (Figure 5F arrows).

Albumin labeling of the sham retina was especially high in most cell bodies in the GCL, INL, and in processes in the IPL (Figure 4G, Figure 5G arrows). The Müller cells and interphotoreceptor matrix (IPM) were labeled weakly but the photoreceptors showed little or no labeling (Figure 4G). Albumin labeling showed a prominent increase in intensity within the detached inner retina (Figure 4H, Figure 5H). At this resolution, albumin appears in the Müller cells (colocalized with vimentin) and other retinal cell types (Figure 4H).

The control experiments showed no specific labeling and the images were essentially black (data not shown).

## Discussion

In this study we undertook a comparative proteomic analysis to elucidate proteins whose expression is influenced by retinal detachment. The proteins identified are involved in a wide variety of processes, including cell metabolism, cell structure, mitochondrial function, and phototransduction, some of which could be used as biomarkers and therapeutic targets.

### Cytoskeletal proteins

The expression of vimentin and tubulin β-2C were found to be significantly changed in the retina following detachment. Vimentin was shown to increase overall on 2D-PAGE analysis, with immunocytochemistry confirming this to be primarily within the Müller cells [12]. Although immunocytochemistry of detached retina showed an increase in tubulin β-2C labeling in the largely surviving inner retina and Müller cells, 2D-PAGE analysis showed an overall decrease in retinal tubulin β-2C following detachment. This overall loss of tubulin β-2C is in agreement with previous reports and is likely attributable to the extensive degeneration and loss of the photoreceptor layer [36]. Indeed, the rabbit retina shows a particularly rapid degeneration of the ONL and outer plexiform layer (OPL) following detachment in comparison to other species, which may in part be due to the lack of intraretinal vasculature [26,37-39]. Beta-tubulin, vimentin, and glial fibrillary acidic protein (GFAP) may structurally reinforce the endfeet region of Müller cells and are upregulated throughout this cell type following retinal detachment and other forms of injury [36,40]. Indeed, in mice lacking GFAP and vimentin, the endfeet layer of the Müller cells can easily separate from the rest of the retina following manipulations such as enucleation and retinal detachment [40,41]. Moreover, the Müller cells in these mice show an altered response to detachment and are unable to hypertrophy outside the retina, reinforcing the view that the intermediate filament proteins structurally support their growth. If Müller cell processes extend into the subretinal space forming subretinal membranes they can impede photoreceptor regeneration even after successful retinal reattachment surgery [18]. Reattachment can stop the continued growth of Müller cell processes into the subretinal space, although they are then induced to grow onto the vitreal retinal surface [42].

### Proteins primarily involved in glycolysis

Four fragments of α-enolase were found to be upregulated following retinal detachment. Western blotting also revealed multiple discreet bands, of which those at the lower molecular mass probably correspond with the 2D-PAGE spots. This rise in α-enolase fragmentation observed at multiple 2D-gel locations may be indicative of extensive post-translational modification, possibly in keeping with the multifunctional nature of this protein that extends beyond glycolysis [43], an increased level of protein degradation, and/or increased synthesis of the full length protein.

Retinal detachment leads to intraretinal hypoxia [44], which may be especially pronounced in the predominantly avascular rabbit retina [39]. This oxygen deprived environment may have caused an increased expression of α-enolase following detachment, in an effort to obtain more energy through glycolysis [45]. Indeed, α-enolase is an abundant cytosolic enzyme in many cell types where it catalyzes the interconversion of 2-phosphoglycerate to phosphoenolpyruvate as part of the glycolytic pathway [46]. Hypoxic-induced upregulation of α-enolase is likely to be significantly mediated by the key regulatory protein hypoxia-inducible factor (HIF)-1, which has a crucial role in several oxygen-dependent diseases of the retina [47,48]. Indeed, in addition to α-enolase, HIF-1 is able to activate the transcription of genes encoding vascular endothelial growth factor, erythropoietin, and other glycolyic enzymes, including fructose-bisphosphate aldolase A, which was also found to be differentially expressed in the present study (discussed below) [47,49-51].

Müller cells have been found to be more resilient to the effects of ischemia and hypoglycaemia in comparison to neuronal cells, probably as a consequence of their high glycogen reserves and unusual preference for glycolysis as the principle source of their energy even under normal aerobic conditions [52,53]. Immunocytochemistry of the sham retina demonstrated particularly high α-enolase labeling of the Müller cell endfeet. This region, which is well known for its significant K^+^ conductance [54], is also postulated to be the origin of the intermediate filament response [27], a factor that may be associated with the possible accumulation of α-enolase that was observed within the Müller cells following retinal detachment in the present study.

Alpha-enolase has also been localized to the cell surface where it serves as a major receptor for plasminogen, facilitating its activation [55]. Aside from fibrinolysis, the plasminogen activators and plasmin system play a role in ECM degradation [56], and are involved in the regulation of matrix metalloproteinases [57], ECM associated growth factors [58], and cell migration [59].

The α-enolase transcript encodes the α-enolase protein with a predicted molecular mass of 47 kDa. However, with the use of a downstream alternative start codon, the α-enolase transcript can be translated into a 37 kDa Myc-binding protein (MBP)-1, which is predominantly localized to the cell nucleus [43,60]. Indeed, the mass spectrometric sequences of all the peptides analyzed were located in the MBP-1 region and we, therefore, cannot differentiate the identified protein as α-enolase or MBP-1 (Appendix 1, Figure 2). Nuclear located MBP-1 is able to bind with the P2 promoter of the c-myc protooncogene, which is an important regulator of apoptosis, cell proliferation, and differentiation [60,61], and thus could play a role in regulating the extensive cellular remodeling that occurs after retinal detachment.

One 2D-gel spot was identified as fructose-bisphosphate aldolase A, a glycoltyic enzyme that catalyzes the conversion of fructose 1,6-bisphosphate into glyceraldehyde 3-phosphate and dihydroxyacetone phosphate. The amount of aldolase A appeared to diminish in the retina following retinal detachment, which may be secondary to the photoreceptor cell loss observed, increased degradation, or a decrease in protein synthesis. The changes in expression could also be related to the ability of aldolase A to influence retinal cell structure and motility through its known binding interactions with the actin component of the cytoskeleton [62-64]. Indeed, the levels of actin in the retina have been shown to decrease following retinal detachment, possibly contributing to destabilization of photoreceptor structure, including outer segment degeneration [36].

### Mitochondrial proteins

The retina is an extremely metabolically active tissue [65,66], with the photoreceptors among the most energetic cells in the body [67]. We observed decreased levels of the two mitochondrial proteins, ATP synthase β and creatine kinase with retinal detachment. These findings are likely to be associated with a decreased need for energy metabolism, and/or the disruption of the photoreceptor ellipsoid region and mitochondria loss known to occur as a consequence of photoreceptor degeneration following retinal detachment [27].

The catalytic activity to produce ATP from ADP and inorganic phosphate by way of a proton gradient is located on the β subunit of mitochondrial ATP synthase [68]. Immunocytochemical studies of ATP synthase β within the sham retina showed especially high labeling in the mitochondrial rich Müller cells and the inner segments of the photoreceptors [69-71]. Following retinal detachment, the Müller cells continued to be brightly labeled, especially in the inner half of the retina, while the photoreceptor inner segments and remaining retina showed a decreased signal. Indeed, the loss of mitochondria from their major localization in the inner segments of the photoreceptors and also their synaptic endings in the OPL due to retinal detachment has been shown to be preventable by simple hyperoxia treatment [71]. The dysfunction and loss of mitochondria are likely to have profound effects on the ability of the highly active photoreceptors to regenerate, despite surgical reattachment of the retina [5].

Mitochondrial creatine kinase (MtCK) was found to be reduced after retinal detachment at two closely positioned 2D-PAGE spots, largely in accordance with the predicted molecular mass. Creatine kinase reversibly catalyzes the phosphorylation of creatine with the use of ATP. The phosphocreatine reservoir that is formed plays a crucial role in cellular ATP buffering and transport, particularly in cells with high and fluctuating energy needs such as those present in the retina [72,73]. MtCK is also able to prevent the formation of reactive oxygen species (ROS) [74], and by directly inhibiting the permeability of the mitochondrial outer membrane it can help to avert mitochondrial metabolic failure [75,76]. Indeed, mitochondrial failure can directly lead to cell necrosis, or by the release of appropriate factors, can trigger apoptosis [77-79]. The degeneration and subsequent death of photoreceptors following retinal detachment is known to occur mainly by apoptosis, with necrosis playing a smaller role [5,80].

### Albumin

Three 2D-gel spots identified as fragments of albumin were found to increase with retinal detachment. Increased protein fragmentation suggests that higher levels of proteolytic cleavage occur in detached retina. Indeed, albumin degradation is known to occur in tissues throughout the body [81]. Using immunocytochemistry, we demonstrated intracellular albumin within most retinal cell types, mainly confined to the inner retina, which increased in intensity with detachment. The source of albumin within the retinal cells is unclear, however, the cellular internalization of albumin is believed to involve a receptor-mediated process [82-84]. It is widely believed that albumin and other proteins from the vitreous can enter the subretinal space through a retinal break, where they become concentrated as water and small molecules are absorbed across the RPE [85]. Indeed, pathological subretinal fluid following retinal detachment has a very different protein composition from normal IPM, containing significant amounts of serum proteins such as albumin, transferrin, and gammaglobulin subunits [86]. Albumin may also originate from the retinal vasculature, the rate of which may presumably be increased by transport mechanisms or following RPE damage [87]. However, no blood was noted during the detachment surgery or tissue harvesting that would indicate damage to the RPE-choroid complex or the blood vessels of the rabbit retina, which is predominantly avascular apart from a band of retinal vessels on either side of the optic disc [26,39]. Nevertheless, it is well known that the integrity of the blood-ocular barrier can be compromised due to the physical and metabolic effects of retinal detachment [3,88-90]. It should also be considered that although it is generally presumed that the liver is the main, if not sole, source of albumin synthesis, extrahepatic production sites such as the brain have been suggested [91,92]. It may, therefore, be possible that the increased presence of albumin fragments observed in the present study could also be explained by de novo synthesis.

Albumin is the most abundant vitreal and plasma protein, where in the latter it is known to function as the key regulator of the colloidal osmotic pressure of blood and as a major transport protein for many important metabolic molecules [81,93,94]. Indeed, vitreal albumin has been suggested to facilitate the transport of long chain fatty acids into the lens for the synthesis of lens lipids [93]. This versatile protein also possesses anti-inflammatory and apoptotic regulatory properties, and is able to maintain redox potentials by scavenging ROS and sequestering redox active transition metal ions [81,95-97]. It may be possible that increased albumin within the retina could result from local synthesis as a protective response to the oxidative stress produced by retinal detachment [98,99], in a manner similar to that which has been proposed for glaucoma [100]. Since albumin has also been localized to the IPM of the retinas of humans and other vertebrate species in accordance with our observations, and has been shown to have the ability to bind with retinoids, it has been postulated that albumin could contribute to the transport of visual cycle molecules, alike to interphotoreceptor retinoid-binding protein [91,101]. Indeed, the role of albumin in healthy and detached retina may be as significant and diverse as its known functions throughout the body, though this remains to be characterized.

### Prohibitin 1

2D-PAGE analysis showed prohibitin 1 to be upregulated following retinal detachment. Prohibitin 1 forms a physiologically active structure by complexing with the closely related protein prohibitin 2 (prohibitone) [102]. These poorly understood proteins are ubiquitously expressed in a wide range of organisms and have been localized to the mitochondrial membrane, and more recently to the cell nucleus and plasma membrane [103]. The prohibitins are postulated to stabilize newly synthesized respiratory chain proteins, maintain mitochondrial structure, repress transcription and cell cycle progression, and regulate apoptosis [102,104-108]. These proteins have been associated with certain pathological states, including inflammation [109] and cancer [110], and may well play an important role in cellular processes following retinal detachment.

### Transducin-β_1_

The level of rod transducin-β1 subunit was shown to decrease following retinal detachment. Transducin is a heterotrimeric (α- β- and γ-subunit) guanine nucleotide-binding protein that is coupled as different isoforms with rod and cone opsin, and functions in the phototransduction cascade [111,112]. Excessive phototransduction signaling and dysfunctional protein trafficking involving transducin has been implicated as potentiating factors of photoreceptor apoptosis in light-induced and inherited retinal degenerations [112-114]. In the present study, however, the downregulation of rod transducin-β_1_ is likely a reflection of the generalized photoreceptor cell degeneration that was observed in the rod-dominated rabbit retina following detachment [26,37,38].

### Conclusions

The differentially expressed proteins elucidated in this study may play an important role in the cellular responses of the retina after its detachment, subsequent ability to recover following surgical reattachment, as well as in serious complications such as subretinal fibrosis and PVR. Further investigations of these proteins are necessary to determine their functions, and to establish their use as potential biomarkers and therapeutic targets.

## Appendix 1. Mass spectrometric identification, including sequence information, of the 2D-PAGE protein spots differentially expressed in the rabbit retina.

Protein spots 5101, 5703, and 8605 with fold changes of 0.48, 0.43, and 0.38 respectively could not be identified. Individual Mascot ions scores greater than approximately 36 indicate identity or extensive homology (p<0.05). Biological processes were taken from the Gene Ontology Consortium. Abbreviation: RD represents retinal detachment. To access the data, click or select the words “Appendix 1.” This will initiate the download of a compressed (pdf) archive that contains the file.
